# Microscopic Examination of a Relaxed Uvula

**Published:** 1852-10

**Authors:** T. Inman

**Affiliations:** Lecturer on Materia Medica, Therapeutics and Medical Jurisprudence, etc.


*Microscopic Examination of a Relaxed Uvula.* By T. Inman, M.D., Lecturer on Materia Medica, Therapeutics, and Medical Jurisprudence, etc. A gentleman, whose father had suffered from a similar affection, was complaining of severe cough for some weeks, apparently arising from relaxation of the uvula.

On examining this appendage during an attempt at deglutition, the upper part was seen to contract firmly, leaving the lower part perfectly smooth and unchanged. It seemed thickened, but its color was normal. I recommended excision, which was at once submitted to, and I procured the fragment for examination. It was very firm, almost fibrous in the interior, and covered with the ordinary mucous membrane, which was not hypertrophied. On making sections of the fibrous part, and tearing them up with needles, the structure was found to consist of groups of large cells, 1-250th inch in diameter, and studded with an immense number of granules. These were probably portions of enlarged and altered mucous glands, and were bound together by strong areolar tissue. Amongst these were interspersed numerous muscular fibres, arranged in bundles. They were 1-750th inch in diameter, but had few of the ordinary characters of voluntary muscle. Very few were striated —all of them seemed to be composed of an outer membrane, and an inner substance closely resembling fibrin. It was homogeneous, yellow, divided irregularly into fragments by transverse divisions. All elasticity seemed to be gone. The majority of the fibres terminated in blunted points, as if they were undergoing absorption.

From this it will be seen that relaxation of the uvula is not (in every case at least) dependent upon muscular debility only, and therefore to be cured by local stimuli, but that it is sometimes occasioned by an actual change of structure preventing contraction. The part so diseased acts as a constant dead weight, and increases the mischief by dragging down that portion which is still healthy. In this case no other remedy but excision could have been of any use. This simple corollary may be deduced from the microscopic appearances, viz., whenever the lower portion of the uvula is quite motionless during deglutition, it is useless to attempt to cure it, except by removal.—*London Medical Times of September* 4, 1852.

